# Growth and neurodevelopmental status in patients with retinopathy of prematurity treated with intravitreal bevacizumab: a case–control study

**DOI:** 10.1186/s40942-021-00340-6

**Published:** 2021-11-16

**Authors:** Majid Abrishami, Hassan Boskabadi, Mojtaba Abrishami, Farid Shekarchian, Majid Khadem-Rezaiyan, Nasser Shoeibi

**Affiliations:** 1 Eye Research Center, Mashhad University of Medical Sciences, Qarani Blvd, 9195965919 Mashhad, Iran; 2 Neonatal Research Center, Mashhad University of Medical Sciences, Mashhad, Iran; 3 Clinical Research Development Unit, Mashhad University of Medical Sciences, Mashhad, Iran

**Keywords:** Retinopathy of prematurity, Growth, Neurodevelopmental status, Bevacizumab, Retina

## Abstract

**Background:**

The current study aimed to evaluate growth and neurodevelopmental status in patients with retinopathy of prematurity (ROP) treated with intravitreal bevacizumab (IVB).

**Methods:**

This historical cohort study was conducted on neonates with ROP who were treated with IVB and age and birth weight-matched controls who did not need IVB. Apgar score less than five, history of blood transfusion and history of infectious diseases were among exclusion criteria. Indirect ophthalmoscopic examinations were performed till complete retinal vascularization. Growth and neurodevelopmental status were evaluated by Age and Stages Questionnaire (ASQ) at the ages of 6, 12, and 18 months. Developmental milestones were assessed in five areas (gross motor, fine motor, personal-social status, problem-solving, and relationship) and overall issues.

**Results:**

A total of 34 cases and 36 controls were included in the present study. Birth weight and corrected gestational age were not statistically different between the groups. In a follow-up period of 18 months, bevacizumab was effective as a primary treatment in the treatment of severe cases of ROP. There was no significant difference between the two groups regarding the five areas and overall issues in follow-up intervals (P > 0.05).

**Conclusions:**

The obtained results did not show any growth and neurodevelopmental differences between treatment-naïve infants and those receiving IVB for the treatment of ROP.

## Background

Retinopathy of prematurity (ROP) accounts for 4% and up to 40% of all cases of childhood blindness in developed and developing countries, respectively [1] The following factors increase the risk of this disease: gestational age of 32 weeks or less, weight of less than 1250 g, intrauterine growth retardation, and exposure to supplemental oxygen for a long time [2, 3].

Retinal vascularization originates from the optic disc at about 16 weeks of gestation [4]. The blood vessels grow gradually toward the periphery of the developing retina to supply oxygen and nutrients. At term gestation, the retinal vessel growth is almost complete, while in preterm neonates the normal pattern of vascularization is disrupted and may be interrupted. The peripheral area of the non-vascularized retina is at risk of oxygen deprivation [5]. Vascular endothelial growth factor (VEGF) is the major growth factor which is responsible for angiogenesis, and high levels of VEGF level is considered to be the primary angiogenic factor mediating retinal neovascularization in eyes with ROP [5]. The studies conducted on patients with stage 3 and 4 ROP demonstrated that the vitreous concentration of VEGF was significantly higher, compared to that in eyes with inactive ROP [6, 7].

Bevacizumab (Avastin; Genentech Inc., South San Francisco, California, USA) as an anti-VEGF has shown the beneficial effect for zone I or II posterior stage 3 with plus disease retinopathy of prematurity [8]. Although intravitreal bevacizumab (IVB) is off-label in ROP treatment, the advantages of IVB for ROP include ongoing retinal vascularization, the short duration of the procedure, and the absence of anesthesia [4, 8]. Nonetheless, bevacizumab enters the general circulation after IVB injection, may suppress plasma VEGF levels, and can be detected in the blood for 8 weeks in patients with type 1 ROP [9–11].

Owing to the necessity of VEGF for normal angiogenesis, as well as its neuroprotective and growth effects, the present study was conducted to assess the developmental status in infants who underwent intravitreal bevacizumab injection for the treatment of ROP.

## Methods

### Study participants

This historical cohort study was performed at Khatam Eye Hospital, a referral ROP center in Northeast Iran. The consecutive patients with a diagnosis of bilateral type 1 ROP who needed bilateral IVB injection (0.625 mg/0.025 mL) confirmed by two retina specialists were included. The exclusion criteria at the time of patient and control selection entailed: (1) a history of intracranial hemorrhage, (2) Apgar score less than five, (3) birth weight more than 1500, (4) gestational age more than 33 weeks, (5) any history of blood transfusion, (6) any history of TORCH syndrome, and (7) other infectious systemic diseases affecting the central nervous system. The gestational age-matched control cohort was comprised of premature neonates who were followed up in the same center and they did not need IVB for retinal vascularization because of of insufficient severity. Detailed ocular and systemic histories were obtained from each subject. The ophthalmic examinations were performed based on a predetermined schedule according to guidelines provided by the American Academy of Pediatrics using RetCam (Clarity Medical Systems, Pleasanton, CA USA) and indirect ophthalmoscopy till complete retinal vascularization reached the ora serrata.

### Ages and stages questionnaire

Growth and development of the selected patients and control group were evaluated by one neonatologist (H.B.) using general examination, past medical history, gestational and birth history, as well as Ages and Stages Questionnaire (ASQ) assessment. Date of birth, height, weight, and head circumference was registered and assessed on every pediatric examination at 6, 12, and/or 18 months of corrected age.

The ASQ, which was used for the assessment of developmental status, is one of the most widely used parent-completed questionnaires that pinpoints developmental progress in children between the ages of 1 month to 5 ½ years [12]. ASQ measures developmental milestones in five areas (communication, fine motor, gross motor, problem solving ability, and personal-social functioning). Parents complete the questionnaire, indicating for each item “yes” if child performs the item, “sometimes” indicating an occasional or emerging skill, or “not yet” indicating that the child does not yet perform the behavior. Responses to the six questions in each area are added to calculate a score for each area. Scores for each area should fall between 0 and 60 (Yes = 10 points, Sometimes = 5 points, Not yet = 0 points). Higher scores indicate more positive outcomes. Each area of the ASQ has different cutoff scores. If the baby’s score in each area and the total score is above the cutoff point, baby’s development is considered to be on schedule. This test performs well with children with prematurity [13, 14]. It has been proven to be a reliable and valid screening test even in its translated and culturally-adapted versions. This questionnaire has been adapted, validated, and standardized in the population studied [15]. The mean score was calculated for score calculation of every aspect due to the different number of items in various aspects of ASQ. Head circumference and weight gain were also included in the assessment. In our study, a neonatologist supervised the filling of questioners by parents.

### Statistical analysis

The normal distribution of variables was examined using the Kolmogorov–Smirnov test. Qualitative variables were expressed using percentages. T-test, ANOVA (or Mann–Whitney’s test and the Kruskal–Wallis test as non-parametric equivalent tests, respectively) and chi-square test were used for inferential statistics. The level of statistical significance was set at 0.05. All statistical analyses were performed using SPSS software (version 11.5) (IBM SPSS Statistics, IBM Corporation, Chicago, IL, USA).

### Ethical considerations

The study protocol adhered to the tenets of the Declaration of Helsinki. Parents of all participants provided written informed consent before enrollment, and the ethical aspects of the study were approved by the Regional Committee on Medical Ethics at Mashhad University of Medical Sciences, Mashhad, Iran (IR.MUMS.REC.1393.78).

## Results

A total of 34 cases (19 males) and 36 gestational age-matched control neonates (20 males) with ROP of insufficient severity to require intervention, were recruited in the study from January 2015 to January 2016. There was no significant difference between the two groups regarding supplemental oxygen therapy (p = 0.064) or duration of neonatal intensive care unit (NICU) stay (p = 0.455). Weight and head circumference data at birth, as well as 6, 12, and 18 months in both groups are presented in Table 1.

**Table 1:** Weight and head circumference exam time points (mean ± standard deviation)

	Exam time point (corrected post-gestational age)			
	Birth	6 month	12 month	18 month
Weight (Kg)				
Case	1.23 ± 0.05	8.03 ± 0.39	9.18 ± 0.24	10.53 ± 0.57
Control	1.35 ± 0.04	7.06 ± 0.21	9.35 ± 0.23	10.45 ± 0.65
P value	0.071	0.043	0.609	0.933
Head circumference (cm)				
Case	26.36 ± 0.62	43.15 ± 0.43	44.59 ± 0.34	45.93 ± 0.69
Control	28.08 ± 0.33	42.42 ± 0.50	45.33 ± 0.26	46.00 ± 1.08
P value	0.027	0.279	0.088	0.960

Gestational age (P = 0.121) and gender (P = 0.584) did not show significant difference between the two groups. The most severe stage of retinopathy was at the postconceptional age of 37.15 ± 4.46 weeks in the treatment group and 38.32 ± 3.35 in the control group (p = 0.230). Other Demographic characteristics of two groups are presented in Table 2.

**Table 2:** Demographic characteristics of groups

Characteristic	Case	Control	P value
Fertilization			
Normal	20 (74%)	21 (75%)	0.591
IVF	7 (26%)	7 (25%)	
Delivery			
Normal	11 (32%)	8 (23%)	0.270
Cesarean	23 (68%)	27 (77%)	
Pregnancy			
Single	16 (47%)	13 (36%)	0.246
Multiple	18 (53%)	23 (64%)	
Gestational age (weeks)	28.79 ± 1.96	29.86 ± 1.93	0.021
NICU stay (days)	26.17 ± 14.47	28.08 ± 15.90	0.455
O_2_ therapy (days)	24.80 ± 15.90	18.00 ± 10.27	0.064

**Fig. 1:**
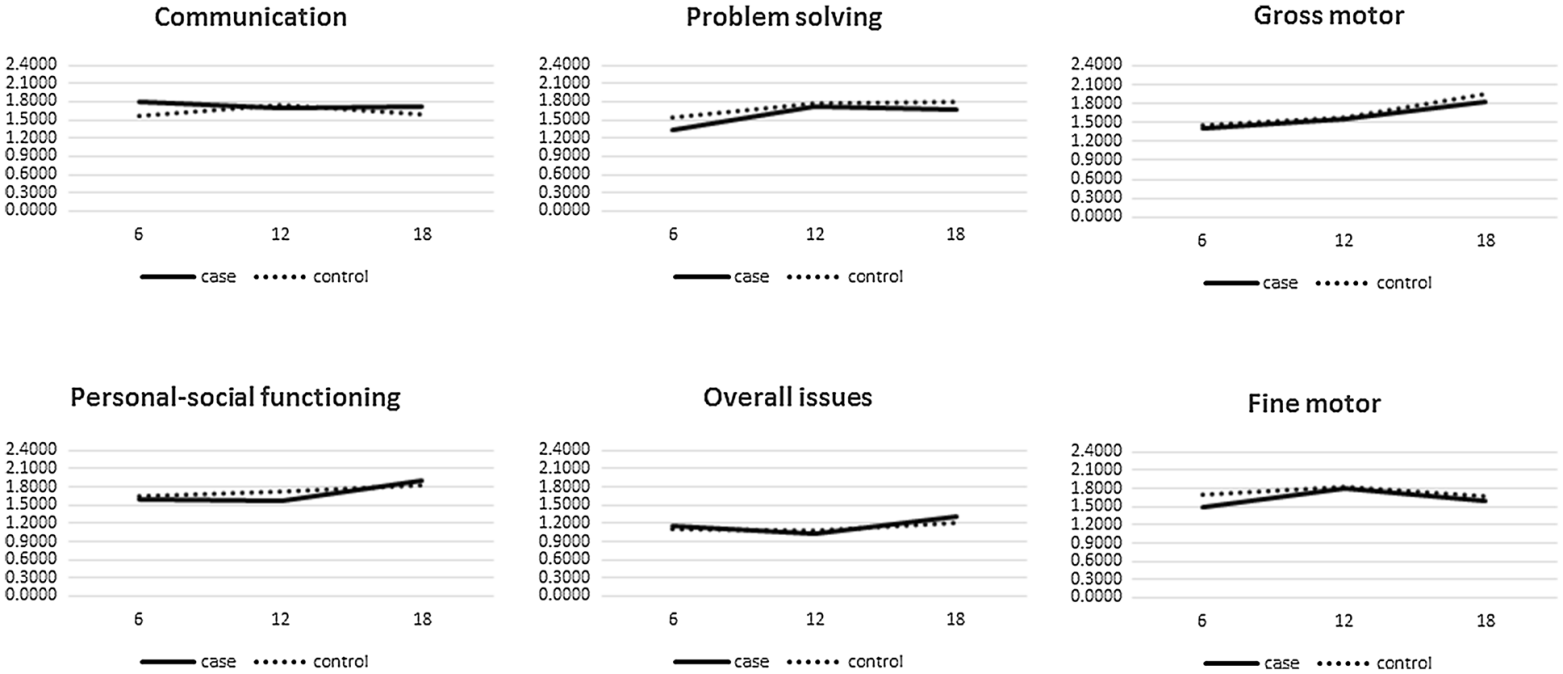
Growth AND development process in six study aspects of ASQ at 6, 12 and 18 month old participants (X axis is the age of study participant and the Y axis is the ASQ score in the related area)

There was no significant difference between the two groups regarding overall ASQ scores. The mean overall scores were obtained 1.16 ± 10.34, 1.03 ± 0.24, and 1.30 ± 0.28 at 6, 12, and 18 months in the case group, respectively. On the other hand, these values were reported as 1.09 ± 0.15, 0.08 ± 0.26, and 1.20 ± 0.31 at 6, 12, and 18 months in the control group, respectively. These scores did not show significant difference; moreover, the scores were not statistically different between cases and controls in the five areas of the questionnaire (Table 3). Time course of measured data in each area of ASQ in both groups are summarized in Fig. 1.

**Table 3:** Mean score of each area of ASQ for different ages in the case and control group

Area	Exam time point (corrected post-gestational age)			P value (between two groups)
	6 month	12 month	18 month	
Communication				
Case	1.80 ± 0.17	1.68 ± 0.41	1.72 ± 0.34	0.168
Control	1.55 ± 0.48	1.74 ± 0.36	1.58 ± 0.46	
P value	0.047	0.594	0.463	
Gross motor				
Case	1.39 ± 0.46	1.55 ± 0.50	1.83 ± 0.39	0.122
Control	1.46 ± 0.47	1.57 ± 0.66	1.94 ± 0.08	
P value	0.691	0.874	0.507	
Fine motor				
Case	1.49 ± 0.52	1.79 ± 0.29	1.58 ± 0.31	0.275
Control	1.69 ± 0.47	1.82 ± 0.28	1.68 ± 0.26	
P value	0.275	0.773	0.520	
Problem solving				
Case	1.33 ± 0.38	1.72 ± 0.37	1.67 ± 0.31	0.469
Control	1.55 ± 0.58	1.77 ± 0.39	1.80 ± 0.16	
P value	0.261	0.684	0.373	
Social-personal				
Case	1.60 ± 0.45	1.58 ± 0.43	1.89 ± 0.08	0.627
Control	1.65 ± 0.48	1.71 ± 0.51	1.83 ± 0.21	
P value	0.795	0.265	0.518	
Overall				
Case	1.16 ± 0.34	1.03 ± 0.24	1.30 ± 0.28	0.512
Control	1.09 ± 0.15	1.08 ± 0.26	1.20 ± 0.31	
P value	0.482	0.476	0.508

## Discussion

The present study assessed neonates with ROP who needed IVB and compared their growth and development status with those without IVB or any intervention. The obtained results did not find any differences between cases and premature neonates without a history of IVB; moreover, the IVB showed no effect on the developmental status till 18 months of age. Furthermore, no difference was observed between the two groups of treatment and control in terms of weight gain and head circumference till 18 months.

In patients with stage 3 ROP, laser photocoagulation and cryotherapy are the current destructive therapies, and visual field constriction is their important complication [8]. The IVB has been introduced as an effective treatment for type 1 ROP. It blocks the VEGF-A function which is shown to be an effective treatment in ROP [7–11].

BEAT-ROP (Bevacizumab Eliminates the Angiogenic Threat of Retinopathy of Prematurity) was a leading study on the effectiveness of bevacizumab in type 1 ROP [16]. There has been more interest in anti-VEGF therapy for ROP since then. However, there are many unanswered questions regarding long-term ocular and systemic adverse effects. The conventional dose of IVB in infants is half to one-fourth of adult dosages, demonstrating the relatively high dose of intravitreal anti-VEGF in preterm neonates [17, 18].

Blood-ocular barrier have been regarded as an effective barrier regarding the penetration of drug from the vitreous into the blood; nevertheless, recent studies have shown the escape of anti-VEGF into the blood circulation. For instance, Miyake et al. indicated that in primates, bevacizumab enters the general circulation, suppresses plasma VEGF levels, and can be detected in the blood for more than 8 weeks [11]. Sato et al. evaluated the level of bevacizumab and serum VEGF after IVB in ROP neonates and reported that the serum level of bevacizumab significantly increased over two weeks after the injection with an inverse decrement in the serum level of VEGF [10].

The inhibition of VEGF bioactivity in preterm neonates may affect normal ongoing growth and development in multiple organs while disrupting normal angiogenesis and the neuronal process since VEGF has been recognized as the survival factor for neuronal and endothelial cells. In the brain, VEGF has neuroprotective and neurotrophic properties; moreover, it helps in the maintenance of the blood–brain barrier, and in the lungs, VEGF plays a key role in alveolar development and surfactant synthesis. It is also critical for glomerular development in kidney and skeletal growth [17, 18]; therefore, the blockage of VEGF may affect different organs of developing neonates.

In a case series of 13 patients who underwent IVB for ROP and were followed up for 5 years, only 1 patient showed a delay in growth and neurodevelopment and the others were within the normal range [19]. In the stated study, Denver Developmental Screening Test II (DDST II) was used to evaluate the growth and the neurodevelopment of the patients. It is noteworthy that no control group was included in the referred study. The results of the current study showed no developmental delay in neonates with ROP who were treated with IVB, as compared to the gestational age-matched control group. Furthermore, the ASQ was used as the screening test which performs well with children with prematurity [13, 14].

Another case series of 137 patients with a history of IVB for ROP treatment assessed the effect of intravitreal anti-VEGFs on neurodevelopment using the developmental-quotient (DQ) score. They followed the patients for 6 years and showed that there were no differences between the study population and the norms for the areas of personal social age, motor adaptive skills, gross motor skills, and language [20]. The same findings are repeated in another study by Chang et al. in a 2 year follow up using Bayley-II assessment tool [21]. In the present study, growth and development were evaluated by ASQ form in five areas (communication, fine motor, gross motor, problem-solving ability, and personal-social functioning). Nonetheless, in the Chang’s study, the areas of personal-social, motor adaptive skills, gross motor skills, and language were compared between the study population and the norms.

Recently, a study conducted by Morin et al. was published based on the data from the Canadian Neonatal Network and the Canadian Neonatal Follow-Up Network databases [22]. Out of 125 treated neonates, 27 cases received bevacizumab, and 98 newborns were treated by indirect laser therapy. The bevacizumab group had higher odds of severe neurodevelopmental disabilities at 18 months corrected age on the basis of the Bayley Scales of Infant and Toddler Development, compared to patients in indirect laser therapy. However, infants treated with bevacizumab appeared sicker upon admission. There was a trend toward a longer neonatal hospitalization and a lower proportion of females in the bevacizumab group. In the present study, there was no significant difference between the bevacizumab group and the treatment-naive controls regarding sex ratio and NICU stay. Furthermore, we excluded the patients with infectious diseases affecting the growth and neurodevelopmental status. In a retrospective study by Arima et al. neurodevelopmental examination was performed using the Kyoto Scale of Psychological Development (KSPD) examination tool. They found that the administration of IVB was significantly associated with neurodevelopmental delay in the Language-Social area but not Postural-Movement, Cognitive-Adaptive areas or overall developmental quotient (DQ) [23].

The major limitation in this study is the relatively short follow up of 18 month. Another drawback in this study is the lack of control group with laser treatment. Of course, attention to the results shows that in most basic and demographic factors, these two groups were matched, therefore, the absence of a laser-treated group will not be a defect for this study. The other shortcoming in this study was the higher gestational age of the control group. However, two groups had no significant difference in developmental scores despite the lower gestational age of treatment group who received IVB. Another defect of this study is low sample size. So it is recommended to perform more studies with higher sample sizes and longer follow ups as well as IQ testing later. Furthermore, assessment of cerebral palsy was not performed in this study. The abovementioned shortcomings should be considered in generalizability of findings in this study.

In conclusion, the results of the current study demonstrated that IVB exerted no effect on the growth and developmental process of ROP children. Nevertheless, it is recommended to perform prospective studies with longer follow-up and larger sample size to determine the long-term effect of intravitreal bevacizumab on systemic and ocular growth and development.

## Data Availability

The datasets generated and analyzed during the current study are available from the corresponding author on reasonable request.

## Abbreviations

ROP: Retinopathy of prematurity
VEGF: Vascular endothelial growth factor
IVB: Intravitreal bevacizumab
ASQ: Ages and stages questionnaire
DDST II: Denver Developmental Screening Test II
DQ: Developmental-quotient
BEAT-ROP: Bevacizumab Eliminates the Angiogenic Threat of Retinopathy of Prematurity
NICU: Neonatal intensive care unit
